# What does it take to assess dementia in community‐dwelling immigrant older adults with limited English proficiency? – A case of Korean Americans

**DOI:** 10.1002/alz.092960

**Published:** 2025-01-09

**Authors:** Hae‐Ra Han

**Affiliations:** ^1^ Johns Hopkins University School of Nursing, Baltimore, MD USA

## Abstract

**Background:**

Currently, there is no national consensus on how to identify individuals with probable dementia in community‐based settings. With the rapid increase of aging populations—particularly ethnic minorities—there is an urgent need to create a process to effectively identify individuals with probable dementia to adequately plan for dementia care. The aim of this study was to evaluate a dementia screening approach applied to a recent immigrant community, Korean Americans.

**Method:**

PLAN—Preparing for healthy aging through dementia Literacy education And Navigation—is an ongoing trial involving community‐dwelling Korean older adults with probable dementia and their care partners in the greater Washington and metropolitan New York areas. Data for the current analysis came from: community coordinator logs, recruitment tracking notes, and participant enrollment database.

**Result:**

The study team developed a two‐phase screening process to ensure accurate assessment of dementia status: (1) Mini‐Mental Status Exam (MMSE) and (2) Clinical Dementia Rating (CDR). The screening protocol was written so once the first‐phase screening meets the pre‐established criterion (MMSE<24), the older adult‐caregiver dyad is invited to a CDR interview to confirm the dementia status. Given lack of bilingual/bicultural geriatric workforce in the community, bilingual community health workers (CHWs) were trained to conduct both screenings. As of January 19, 2024, trained CHWs screened 2,693 older adults using MMSE; 584 of them (21.7%) were screened positive and invited to the second‐phase screening using CDR. More than two thirds (n = 392 or 67.1%) agreed and participated in CDR assessment; 309 of them (or 78.8%) received a score of 1+, indicating probable dementia.

**Conclusion:**

We were able to train bilingual CHWs to conduct dementia screenings in one of the most understudied and underserved Asian immigrant communities. The prevalence of dementia was more than 20% in the screened sample, compared to 6%∼13% in the general US older population. Nearly four of five among the MMSE positive sample were confirmed to have a substantial functional decline based on CDR, indicative of probable dementia. When working with resource‐poor communities, our approach of mobilizing community assets (CHWs) and combining a brief cognitive screener with a refined functional measure can help identify older adults with probable dementia.

